# Once-Weekly Semaglutide Improves Body Composition in Spanish Obese Adults with Type 2 Diabetes: A 48-Week Prospective Real-Life Study

**DOI:** 10.3390/jcm14155434

**Published:** 2025-08-01

**Authors:** Irene Caballero-Mateos, Cristóbal Morales-Portillo, Beatriz González Aguilera

**Affiliations:** Endocrinology and Nutrition Department, Vithas Hospital, 41009 Sevilla, Spain; cr.morales@hotmail.com (C.M.-P.); beag141186@hotmail.com (B.G.A.)

**Keywords:** semaglutide, real-world, body composition, type 2 diabetes, fat mass, fat-free mass

## Abstract

**Objective:** The objective of this study was to assess changes in body composition, with a specific focus on fat mass (FM) and fat-free mass (FFM), in obese adults with type 2 diabetes (T2D) treated with once-weekly (OW) subcutaneous (s.c.) semaglutide. **Methods:** This was a single-center, 12-month, real-world, ambispective study (6-month prospective and 6-month retrospective). Body composition parameters were assessed via segmental multifrequency bioelectrical impedance analysis (SMF-BIA). **Results:** A total of 117 patients with DM2, with a median age of 56 years, a median HbA1c level of 9.4%, and a median body weight of 102.5 kg, were included in the study. The median body weight, body fat mass, and visceral fat significantly decreased at 6 months, with values of −9.3, −7.5, and −1.8 kg, respectively. There were further reductions from 6 to 12 months, albeit at a slower rate. The median skeletal muscle mass significantly decreased at 6 months (−1.2 kg), although no further significant reductions were observed at 12 months. **Conclusions:** OW s.c. semaglutide for 12 months significantly improved body composition parameters, mainly at the expense of fat mass loss, with the preservation of skeletal muscle mass. These changes are clinically meaningful, since they impact general metabolic health and are associated with improvements in metabolic control and clinical parameters associated with renal and CV risks, as well as presumable improvements in quality of life.

## 1. Introduction

The prevalence of type 2 diabetes (T2D), which is associated with obesity, has increased dramatically worldwide over the past three decades, reaching pandemic levels and posing a major global healthcare problem [1,2]. Obesity contributes to T2D development by increasing visceral adiposity, which causes inflammation-induced insulin resistance [3,4]. Similarly, T2D and obesity lead to the development of cardiovascular (CV) disease, increasing the risk of CV mortality [5,6]. Consequently, most guidelines for the management of T2D have endorsed a multifactorial and holistic approach with the aim of preventing or delaying CV complications and have highlighted the clinical importance of weight reduction as a pillar of this holistic approach, thus contributing to improving glycemic control [7,8,9,10].

Glucagon-like peptide-1 receptor agonists (GLP-1 RAs) are an effective class of glucose-lowering agents that exert their action by enhancing glucose-dependent insulin secretion, suppressing glucagon, and delaying gastric emptying, which contributes to increased satiety and weight reduction [11,12]; given these pleiotropic effects, GLP-1 RAs have been positioned not only as antidiabetic agents but also as potential therapeutic tools in the management of obesity and cardiometabolic risk [13,14,15].

Among these agents, semaglutide—a long-acting GLP-1 RA—has shown particularly robust efficacy in both glycemic control and body weight reduction. This is evidenced by the findings of the SUSTAIN clinical trial program, where once-weekly subcutaneous semaglutide demonstrated superiority over placebo and other active comparators, such as basal insulin, SGLT2 inhibitors, and other GLP-1 RAs, in terms of HbA1c reduction, weight loss, and cardiovascular risk improvement [16,17,18]. Several studies have evaluated the effects of other GLP-1 receptor agonists and anti-obesity medications on body composition. Liraglutide 3.0 mg, for instance, has been associated with significant reductions in fat mass, although accompanied by modest lean mass loss in some cases [19]. Dulaglutide has also shown beneficial effects, promoting fat mass reduction while partially preserving skeletal muscle mass in patients with type 2 diabetes mellitus (T2D) [20]. Among anti-obesity drugs, orlistat has demonstrated sustained weight loss, primarily at the expense of fat mass, with minimal impact on fat-free mass [21]. Additionally, sodium-glucose cotransporter-2 inhibitors (SGLT2i), such as dapagliflozin, have been associated with reductions in both fat mass and, to a lesser extent, lean mass [22]. These findings underscore the need to further evaluate the body composition profile of semaglutide in real-world clinical practice, particularly over extended treatment periods. Furthermore, the effectiveness of once-weekly semaglutide in achieving glycemic control and reducing body weight has been reported in several studies conducted in real-world clinical settings. However, body weight reduction alone does not discriminate accurately between body fat mass and lean mass or between visceral and subcutaneous fat distribution, which are clinical parameters that have been shown to be associated with changes in cardiometabolic risk [23,24,25]. More specifically, excess fat mass has been linked to inflammation, atherosclerosis, dyslipidemia, and hypertension development [26], whereas skeletal muscle mass preservation has been associated with improvements in insulin sensitivity and increased glucose uptake [27]. Sarcopenia, which is characterized by age-related skeletal muscle mass loss along with impaired muscle strength and function, has been identified as a complication of T2D [28,29,30]. Furthermore, antidiabetic drugs have diverse effects on skeletal muscle mass and strength [31,32].

Few studies have addressed the effects of semaglutide on body composition. Notably, lean mass loss was observed in long-term semaglutide RCTs, such as SUSTAIN 8 [33] and STEP 1 [34], whereas it seemed to be preserved following shorter semaglutide administration in real-life studies [35,36,37,38,39,40].

However, real-world studies assessing the effects of semaglutide on body composition and metabolic outcomes over periods longer than six months are scarce [36]. In this context, the objective of the present study was to evaluate the effects of once-weekly semaglutide on body composition, with a focus on fat mass (FM), visceral adipose tissue (VAT), and skeletal muscle mass (SMM) changes, assessed by segmental multifrequency bioelectrical impedance analysis (SMF-BIA), in a cohort of patients with T2D followed-up for 48 consecutive weeks in a real-life setting.

## 2. Materials and Methods

### 2.1. Design

This was an ancillary study of a real-world, observational, 12-month, ambispective (retrospective and prospective), multicenter study, the Sema-RW study, conducted in 10 tertiary hospitals in Spain, whose primary objective was to assess changes in glycated hemoglobin (HbA1c) and body weight (BW) and whose main results have been recently published [41]. The present study, which focused on body composition parameters, was formally planned in the study protocol and was conducted at a single investigational site (Hospital Virgen de la Macarena, Sevilla, Spain). The study was reviewed and approved by the Ethics Committee of the hospital, and written informed consent was obtained from all participants.

Eligibility criteria for inclusion in the parent study were applied consistently at the subsite level. Adult patients (≥18 years) with a confirmed diagnosis of type 2 diabetes (T2D) who were initiated on once-weekly subcutaneous semaglutide in routine clinical practice for at least 6 months, between June and July 2019 and July 2020, and had accessible clinical records at baseline and at least one follow-up visit (6 or 12 months) were considered eligible. Additionally, they should have at least one measurement of HbA1c, blood pressure, or body weight within ±1 month of the first semaglutide prescription. The study patients might have been previously treated with other hypoglycemic agents, including GLP-1 RA, with the exception of semaglutide. Exclusion criteria included diagnosis of type 1 diabetes, participation in interventional clinical trials involving semaglutide or other investigational agents during the study period, and incomplete medical records that precluded adequate outcome assessment. These criteria ensured alignment with the methodological framework of the SEMA-RW study while allowing for the focused evaluation of body composition outcomes at the single-site level.

The ambispective design consisted of a 6-month retrospective phase prior to the date of informed consent signature, during which clinical data were collected from medical records. This was followed by a 6-month prospective follow-up period, during which data were recorded according to routine clinical practice. Patients initiated treatment with semaglutide at the baseline visit (T0), at which point clinical and body composition variables were recorded. Subsequently, data corresponding to the 6-month timepoint (T6) were collected retrospectively at the time of informed consent, while data at 12 months (T12) were recorded prospectively during follow-up visits, in accordance with routine clinical practice.

### 2.2. Aims and Research Questions

The study aims were the following: (1) to evaluate the long-term effects of semaglutide OW s.c. on body composition in obese adults with type 2 diabetes in a real-life clinical setting; (2) to assess the magnitude and progression of changes in fat mass, fat-free mass, and skeletal muscle mass over a 12-month treatment period; (3) to determine whether semaglutide-induced weight loss predominantly reflects reductions in fat mass versus lean mass; and (4) to explore the association between body composition changes and improvements in metabolic, renal, and cardiovascular risk markers. Additionally, we pursued some research questions, such as the necessary time to observe significant improvement in body composition parameters with semaglutide OW s.c.; whether these improvements are associated with changes in metabolic control parameters (e.g., HbA1c, lipid profile, liver enzymes, and renal markers); whether reductions in body weight are primarily attributable to fat mass loss rather than lean mass loss; and, finally, whether skeletal muscle mass will be preserved during long-term semaglutide therapy in a real-world population.

### 2.3. Study Endpoints and Outcome Measures

The primary efficacy endpoint was the mean change in body composition, with a specific focus on fat mass (FM) and fat-free mass (FFM) from baseline (T0) to 6 months (T6) and 12 months (T12).

The secondary endpoints included mean changes in anthropometric parameters, such as BW, body mass index (BMI), waist circumference, and mean changes in HbA1c throughout the entire observation period (T0, T6, and T12). A reduction in body weight of at least 5% from baseline to 6 and 12 months was considered clinically significant (Table 1).

At baseline (T0), the following study variables were collected: age; sex; relevant medical history and disease status (T2D, diabetic retinopathy, dyslipidemia, chronic kidney disease, metabolic dysfunction-associated steatotic liver disease [MASLD], cardiovascular disease, and congestive heart failure); physical examination variables (blood pressure (mmHg), heart rate (heart rate (heart rate) per minute), waist circumference (cm), body weight (kg), and body mass index); analytical variables (fasting plasma glucose (FPG) (mg/dL), HbA1c (%), albuminuria (mg/g), the urine albumin to creatinine ratio (UACR) (mg/g), the estimated glomerular filtration rate (eGFR) (mL/min/1.73 m^2^), total cholesterol (mg/dL), HDL-cholesterol (mg/dL), LDL-cholesterol (mg/dL), triglycerides (TGs) (mg/dL), uric acid (mg/dL), aspartate transaminase (GOT) (U/L), alanine transaminase (GPT) (U/L), platelet count, creatinine (mg/dL), and fibrosis-4 score (FIB-4)); and semaglutide dosage (mg/dL)), prior to and concomitant with treatment with GLP-1.

In addition, changes in HbA1c, body weight, BMI, FBG, eGFR, the UACR, albumin, creatinine, uric acid, total cholesterol, HDL cholesterol, LDL cholesterol, TGs, AST, ALT, and FIB-4 were evaluated at 6 months (T6) and 12 months (T12).

Body composition was assessed via multifrequency bioelectrical impedance analysis (MF-BIA; Inbody 770, Inbody Co., Ltd., Seoul, Republic of Korea). Following standardized procedures, measurements were obtained with patients fasting for 8 h in a supine position, with each leg at an angle of 45° and each arm at an angle of 30° from the trunk. Body impedance was measured using an alternating current of 100 µA, with frequencies ranging from 1 to 500 kHz. PhA, expressed in degrees, was performed at a single frequency of 50 kHz according to international standards.

The following body composition parameters were determined at baseline (T0), 6 months (T6), and 12 months (T12): visceral fat (VF), fat mass (FM), fat mass index (FMI), fat-free mass (FFM), fat-free mass index (FFMI), skeletal muscle mass (SMM), skeletal muscle index (SMI), phase angle (PhA), total body water (TBW), extracellular water (ECW), and the ECW/TBW ratio. The definitions of the body composition parameters measured by BIA in the present study are displayed in Supplementary Table S1. Changes in body composition parameters between T6 and T0 and between T12 and T0 were calculated.

For safety evaluation, adverse events (AEs) and serious adverse events (SAEs) were collected from medical records during the retrospective phase. The overall study safety results have already been published [35,41], and hence, they were not an objective of this ancillary study.

### 2.4. Statistical Analysis

No formal sample size calculation was performed in this ancillary study. The final number of selected patients to be enrolled was determined based on the availability of subjects at the site.

Quantitative variables are expressed as the means (standard deviations [SDs]) or medians (interquartile ranges [IQRs]) according to parametric and nonparametric distributions, respectively, and qualitative variables are presented as absolute (*n*) and relative (%) frequencies. All analyses were conducted using all patients included in the study via a visitwise approach. Comparisons of the study variables with the baseline variables at each time point were performed via the Wilcoxon matched-pairs signed-rank test. All the statistical analyses were performed via IBM SPSS Statistics version 20.0.0.

## 3. Results

### 3.1. Patient Characteristics

One hundred seventeen patients with T2D from the SEMA-RW study cohort were enrolled in the BIA ancillary study. The median (IQR) age was 56.0 (45.5, 64.5) years, and 50 (42.7%) patients were female. In addition, the median (IQR) baseline values for HbA1c and body weight were 9.40% (7.90, 11.20) and 102.5 kg (93.2, 116.1), respectively; the other baseline anthropometric variables were waist circumference (median (IQR) of 124 cm (115, 132)) and height (median (IQR) of 167 cm (160, 173)). The most commonly recorded comorbidities were hypertension (*n* = 88, 75.2%), dyslipidemia (*n* = 81, 69.2%), and obstructive sleep apnea–hypopnea syndrome (*n* = 22, 18.8%). In addition, 23.3% and 20.4% of the participants were current and former smokers, respectively. The baseline demographic and clinical characteristics of the patients are shown in Table 2.

**Figure 1:**
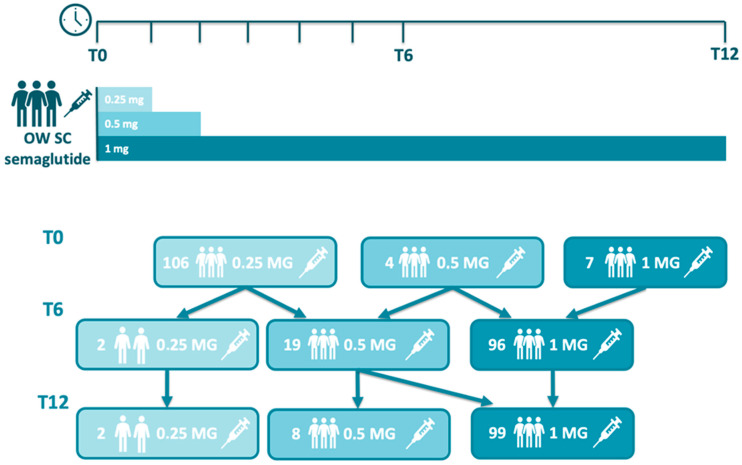
Semaglutide usual dosing and adjustment in the study.

According to the product label, the recommended dosing regimen for once-weekly subcutaneous semaglutide involves an initial dose of 0.25 mg for the first 4 weeks, followed by an increase to 0.5 mg for another 4 weeks, and a further escalation to the maintenance dose of 1.0 mg, depending on tolerability and clinical response. In our cohort, however, the starting doses varied: 106, 4, and 7 patients initiated semaglutide at 0.25 mg, 0.5 mg, and 1.0 mg, respectively. This variation was primarily due to patients switching from other GLP-1 receptor agonists, which allowed for a more rapid titration or direct initiation at higher doses based on prior tolerance. At months 6, 2, 19, and 96, patients were treated with 0.25 mg, 0.5 mg, and 1 mg of once-weekly semaglutide, respectively. Similarly, at months 12, 2, 8, and 99, patients received 0.25 mg, 0.5 mg, and 1 mg of once-weekly semaglutide, respectively, while seven patients discontinued semaglutide therapy. These changes reflect real-world variability in dose escalation based on individualized patient responses and clinical discretion (Figure 1). With respect to the use of concomitant medication (Table 3), when treatment with once-weekly semaglutide was started, 45 (38.5%) subjects were treated with basal insulin, 25 (21.4%) with bolus insulin, 83 (70.9%) with metformin, 32 (27.3%) with SGLT2 inhibitors, and 10 (8.5%) with a GLP-1 RA other than semaglutide and who had switched to semaglutide prior to their enrollment in the study.

### 3.2. Changes in Body Composition Parameters

Overall, most changes in body composition parameters, as assessed via MF-BIA, showed significant reductions at six months (T6) and further reductions at twelve months (T12) versus baseline (Table 4).

Fat mass was significantly reduced at 6 months (median change of −7.6 kg; *p* < 0.001) and 12 months (median change of −9.7 kg; *p* < 0.001). Consistent with these changes, a significant decrease in the visceral fat area was observed at 6 months (median change of −32.9 cm^2^; *p* < 0.001) and at 12 months (median change of −37.7 cm^2^; *p* < 0.001), compared with baseline (median value of 240.1 cm^2^).

Compared with those at baseline, fat-free mass, in turn, significantly decreased at 6 months (median change of −1.80 kg; *p* < 0.001) and 12 months (median change of −2.20 kg). Similarly, the fat-free mass index was significantly lower at 6 months (−0.63 kg/m^2^; *p* < 0.01) and 12 months (−0.72 kg/m^2^; *p* < 0.01) than at baseline. However, no significant changes in either fat-free mass or fat-free mass index at 12 months versus 6 months were observed.

Interestingly, skeletal muscle mass significantly decreased at 6 months (median change of −1.2 kg; *p* < 0.001), compared with the baseline median value of 31.85 kg, although no further significant reductions were observed at 12 months. Furthermore, although the skeletal muscle index significantly decreased at 6 months and continued to decrease at 12 months, most changes occurred within the first 6 months from T0 to T6.

The changes in the extracellular water content were similar to those in the abovementioned body composition parameters. Significant median reductions versus baseline values were observed at 6 months (−0.4 L; *p* < 0.001) and 12 months (−0.9 L; *p* < 0.001), albeit without significant differences between T6 and T12. Nonetheless, no significant variations were observed in the ratio of extracellular water to total body water (ECW/TBW) throughout the follow-up period. Similarly, no significant changes were observed in the phase angle recorded by BIA.

### 3.3. Other Clinical Results

The median changes from baseline in HbA1c levels at 6 (T6) and 12 months (T12) were −3.1% and −3.7%, respectively (*p* < 0.001). Similarly, the median changes from baseline in body weight were −9.3 kg and −11.7 kg at 6 and 12 months, respectively, which were statistically significant (*p* < 0.001). Consistent with body weight modifications, significant reductions in BMI were observed at 6 (−3.7 kg/m^2^) and 12 months (−4.2 kg/m^2^) (*p* < 0.001).

Additionally, some laboratory metrics improved during the entire follow-up period. In addition to HbA1c reduction, significant median changes from baseline at 12 months were found in FBG (−96.0 mg/dL; *p* < 0.001), UACR (−5.1 mg/g; *p* = 0.042), uric acid (−1.7 mg/dL; *p* = 0.03), TC (−15.0 mg/dL; *p* < 0.001), HDL-C (4.0 mg/dL; *p* < 0.001), TG (−43.0 mg/dL; *p* < 0.001), SGPT (−11.0 U/L; *p* < 0.001), SGOT (−10.5 U/L; *p* < 0.001), SGOT), and FIB-4 (−0.16; *p* = 0.027) (Table 5).

## 4. Discussion

As a primary objective, our ancillary study explored long-term body composition changes with a focus on body fat mass and fat-free mass in a cohort of patients with type 2 diabetes who received once-weekly semaglutide treatment. Variations in body composition parameters during the follow-up period were assessed via MF-BIA. This is an accessible, noninvasive, reliable, and accurate technique [42,43] that has been shown to be an optimal and valid alternative to DXA in studies evaluating body composition changes in large groups of patients diagnosed with T2D [44,45].

Consistent with the body weight and BMI reductions observed during the study, body fat mass, fat mass index, and visceral fat significantly decreased at 6 months, which was sustained at a slower rate from 6 to 12 months. Similarly, there were significant reductions in fat-free mass and fat-free mass index at six months, which were also maintained without further decreases until the end of the study. However, the absolute magnitude of fat-free mass and fat-free mass index reductions was lower than the respective fat mass and fat mass index values, which was compatible with the assumption that the contribution of fat mass changes to body weight loss was greater than that of fat-free mass. These findings are in accordance with prior real-world observational studies evaluating the effects of once-weekly semaglutide on body composition in T2D patients, despite being limited to a six-month follow-up [35,37,40]. Notably, a recently published prospective observational study that assessed once-weekly semaglutide use in individuals with T2D for 12 months reported a significant reduction in lean body mass at the end of the study follow-up [35,36], which might suggest a relevant and greater contribution of fat-free mass loss to the changes in total body weight, compared with our study. In fact, although we observed a slight decrease in skeletal muscle mass (assessed through skeletal muscle mass and the skeletal muscle mass index) at 6 months, no further reductions were recorded beyond that time point or until the end of the study, which is consistent with the protective effect of semaglutide in preventing sarcopenia onset. This protective effect of semaglutide has been previously reported, although it is more frequently associated with the oral formulation of the drug than with the subcutaneous formulation [37,38,39].

With respect to the observed changes in water composition, there was a reduction in extracellular water and stabilization of the ECW/TBW ratio, which could have several implications for health. A reduction in extracellular water has been associated with improved body fluid balance, reduced inflammation, and a significant contribution to overall weight loss [46,47]. These effects, in turn, would result in some benefits, such as a reduction in the burden associated with weight-bearing joints, increased glycemic control and insulin sensitivity, CV and renal function improvement, and consequently, a decrease in CV disease risk [48]. Furthermore, a balanced and stable ECW/TBW ratio is critical for preserving normal CV and renal functions and maintaining muscular mass and strength, particularly in the context of weight loss [49,50].

Finally, we did not observe any significant changes in the phase angle value throughout the duration of the present study, a finding that has also been associated with muscle strength preservation [51].

Although phase angle (PhA) and extracellular-to-total body water ratio (ECW/TBW) are not yet widely used in clinical management of patients with overweightness or obesity, their assessment via segmental multifrequency bioelectrical impedance (SMF-BIA) can offer valuable insights into cellular health, fluid distribution, and metabolic risk. In individuals with obesity, PhA tends to be lower—likely reflecting reduced cellular integrity, increased extracellular fluid, and chronic subclinical inflammation. A higher PhA correlates with greater fat-free mass (FFM), better muscle quality, and improved metabolic parameters [52]. Similarly, ECW/TBW inversely correlates with PhA and is associated with inflammatory status and interstitial fluid shifts [53]. Although PhA’s role in obese populations is still emerging, these parameters may help detect early cellular dysfunction or unfavorable fluid balance, even before overt clinical changes. In our study, the minimal changes observed in PhA and ECW/TBW over 12 months suggest maintained cellular integrity and fluid homeostasis during semaglutide therapy, despite significant fat mass reductions. Future research should clarify their practical utility in risk stratification and monitoring of interventions in individuals with obesity.

In addition, consistent with an overall improvement in the cardiometabolic risk profile, several significant changes in analytical parameters (glycemic control, lipid profile, uric acid, UACR, transaminases, and FIB-4) were observed.

The observed reduction in fat mass, along with partial preservation of fat-free mass, is clinically meaningful in the context of T2D. Excess adiposity, particularly visceral fat, contributes to insulin resistance, systemic inflammation, and increased cardiometabolic risk. Therefore, a selective reduction in fat mass, as observed in this study, may lead to improvements in insulin sensitivity, lipid profile, and blood pressure. Additionally, maintaining lean mass is essential to preserve physical function, metabolic rate, and quality of life in individuals with obesity. The observed improvements in HbA1c and other metabolic markers further support the role of semaglutide in improving not only glycemic control but also broader aspects of metabolic health.

With regard to the semaglutide dosing we used in our study, it is important to note that, in clinical practice, it is typically individualized based on tolerability, glycemic control, and prior exposure to GLP-1 receptor agonists. According to the product label, semaglutide is initiated at 0.25 mg weekly for 4 weeks, then increased to 0.5 mg, and, if well tolerated, escalated to a maintenance dose of 1.0 mg. However, in our cohort, some patients started directly at 0.5 mg or 1.0 mg due to previous treatment with other GLP-1 RAs, allowing for more rapid titration. This approach reflects routine clinical decision-making and aims to optimize efficacy while considering individual patient profiles.

To the best of our knowledge, our study and the study by Pantanetti et al. [36] are the only studies evaluating body composition in T2D patients treated with semaglutide for a period of 12 months, allowing the confirmation of short-term improvements in many parameters, as well as their stabilization over time.

However, this study has several limitations. In addition to its observational design and the lack of a control group, which has already been mentioned in the full study publication [41], there are few limitations related to the characteristics of the design of this ancillary study. The sample size for this study was not properly calculated but was affected by the availability of patients from a single site of the SEMA-RW study. Additionally, no information regarding lifestyle interventions was systematically recorded, despite the synergistic effect of dietary changes, increased physical activity, and other lifestyle modifications with GLP-1 RA on body composition, which is well established [54]. Furthermore, given the observational and non-randomized nature of the study, residual confounding and selection bias cannot be fully excluded. Although this was a real-world study, this ancillary study was conducted in a single center, limiting the generalizability of the results. Likewise, the lack of detailed sociodemographic data, since this information was not systematically collected in our study, might further limit the generalizability of our results to more diverse or international populations.

It is worth mentioning that body composition was assessed using multifrequency bioelectrical impedance analysis (MF-BIA), a non-invasive, accessible, and widely used method in routine clinical practice. While MF-BIA offers practical advantages for longitudinal monitoring in real-world settings, it has some limitations. Its accuracy can be influenced by hydration status, recent food intake, physical activity, and disease state, and it may not provide the same level of precision as more advanced imaging techniques such as dual-energy X-ray absorptiometry (DEXA). Therefore, results related to body composition should be interpreted in the context of these methodological constraints.

Recent evidence has strengthened the understanding of how different body composition parameters relate to cardiovascular risk and long-term health outcomes. Beyond the limitations of BMI, metrics such as body fat percentage, fat-free mass (FFM), and waist-to-height ratio (WHtR) have demonstrated stronger associations with cardiovascular disease (CVD), metabolic derangements, and mortality. Studies have shown that excess adiposity, particularly visceral fat, correlates with insulin resistance, dyslipidemia, hypertension, and inflammatory processes that increase CVD risk [55,56]. Notably, fat gain and reductions in FFM over time have been independently associated with a higher incidence of cardiovascular events, especially in middle-aged and older adults [56]. Furthermore, the impact of modifying body composition has been substantiated by longitudinal data: for instance, improvements in FFM or reductions in fat mass are linked to lower risk of all-cause mortality and major cardiovascular events, particularly among individuals with coronary artery disease undergoing rehabilitation [57,58]. These findings support that targeted interventions aiming to improve body composition—either by reducing fat mass or preserving/increasing lean mass—may contribute significantly to lowering cardiovascular and metabolic risks and, by extension, improving overall prognosis and quality of life.

## 5. Conclusions

In this real-life study conducted in patients with T2D, 12 months of treatment with once-weekly semaglutide led to clinically meaningful improvements in body composition, primarily through a significant reduction in fat mass while preserving skeletal muscle mass. These favorable changes were not merely anthropometric; they were accompanied by marked improvements in glycemic control and in clinical parameters associated with cardiovascular and renal risks. Taken together, the findings highlight the potential of semaglutide, not only as a glucose-lowering agent, but as a therapeutic strategy capable of modifying cardiometabolic risk and potentially improving long-term outcomes and quality of life.

## Figures and Tables

**Table 1:** Objectives and endpoints.

Primary Objectives	Primary Endpoints
To assess the long-term effects of OW s.c semaglutide on body composition in T2D obese adults.	Variation in body composition from baseline (T0) to 6 months (T6) and 12 months (T12): FM, FMI, FFM, FMI, SMM, SMI, VAT, ECW, PhA
	Variation in anthropometric parameters throughout T0, T6, and T12: BW, BMI, waist circumference
**Secondary Objective**	**Secondary Endpoints**
To assess whether semaglutide-induced weight loss predominantly reflects reductions in fat mass versus lean mass.	Comparation of FFM vs. FM throughout T0, T6, and T12.
To explore the association between body composition changes and improvements in metabolic, renal, and cardiovascular risk markers.	Variations in different analytical parameters throughout T0, T6, and T12: HbA1c, FBG, eGFR, UACR, albumin, creatinine, uric acid, total cholesterol, HDL, LDL, triglycerides, SGPT, SGOT, FIB-4.

BMI, Body mass index; BW, body weight; ECW, extracellular water; FFM: fat-free mass; eGFR, estimated glomerular filtration rate; FBG, fasting blood glucose; FFMI, fat-free mass index; FIB-4, fibrosis-4 score; FM, fat mass; FMI, fat mass index; HbA1c, glycosylated hemoglobin; HDL, high-density lipoprotein; LDL, low-density lipoprotein; OW, once-weekly; PhA, phase angle; s.c, subcutaneous; SGOT, serum glutamic-oxaloacetic transaminase; SGPT, serum glutamate pyruvate transaminase; SMM, skeletal muscle mass; T2D, type 2 diabetes; TG, triglycerides; UACR, urine albumin-to-creatinine ratio; VAT, visceral adipose tissue.

**Table 2:** Baseline demographic and clinical data.

Baseline Demographic Variables	N = 117
Age, years, median (IQR)	56.0 (45.5, 64.5)
Gender, Female, *n* (%)	50 (42.7)
Weight, kg, median (IQR)	102.5 (93.2, 116.1)
Height, cm, median (IQR)	167 (160, 173)
BMI, median (IQR)	37.1 (33.2, 42.0)
Waist circumference, cm, median (IQR)	124 (115, 132)
Years of evolution of T2D, median (IQR)	3.0 (0.0, 9.5)
Diabetic retinopathy, *n* (%)	4 * (3.4)
Hypertension, *n* (%)	88 (75.2)
Dyslipidemia, *n* (%)	81 (69.2)
SAHS, *n* (%)	22 (18.8)
Chronic kidney disease, *n* (%)	11 (9.4)
NAFLD, *n* (%)	16 (13.7)
Ischemic cardiovascular disease, *n* (%)	5 (4.3)
Ischemic stroke/TIA, *n* (%)	0 (0)
Peripheral artery disease, *n* (%)	4 (3.4)
Heart failure, *n* (%)	3 (2.6)
Smoking	
- Current smoker, *n*/N (%)	24/103 (23.3)
- Former smoker, *n*/N (%)	21/103 (20.4)
- Never	58/103 (56.3)
HbA1c (%), median (IQR)	9.40 (7.90. 11.20)
FBG (mg/dL), median (IQR)	206.5 (148.5, 257.8)
eGFR (mL/min/1.73 m^2^), median (IQR)	99.0 (81.0, 110.0)
UACR (mg/g), median (IQR)	9.20 (4.7, 45.3)
Albumin (g/dL), median (IQR)	4.3 (1.7, 4.7)
Creatinine (mg/dL), median (IQR)	0.8 (0.6, 0.9)
Uric acid (mg/dL), median (IQR)	5.1 (4.2; 6.0)
Total cholesterol (mg/dL), median (IQR)	184.5 (161.5, 208.3)
HDL (mg/dL), median (IQR)	40.5 (34.0. 49.7)
LDL (mg/dL), median (IQR)	101.0 (84.0, 126.0)
Triglycerides (mg/dL), median (IQR)	183.0 (135.5, 265.0)
SGPT (U/L), median (IQR)	30.0 (21.0, 54.0)
SGOT (U/L), median (IQR)	28.5 (19.7, 41.2)
FIB-4 SGPT, median (IQR)	1.0 (0.7, 1.3)

* All non-proliferative cells. BMI, body mass index; CKD, chronic kidney disease; T2D, type 2 diabetes; eGFR, estimated glomerular filtration rate; FBG, fasting blood glucose; FIB-4, fibrosis-4 score; HbA1c, glycosylated hemoglobin; HDL, high-density lipoprotein; IQR, interquartile range; LDL, low-density lipoprotein; NAFLD, nonalcoholic fatty liver disease; SAHS, sleep apnea–hypopnea syndrome; SGOT, serum glutamic-oxaloacetic transaminase; SGPT, serum glutamate pyruvate transaminase; TIA, transient ischemic attack; UACR, urine albumin-to-creatinine ratio.

**Table 3:** Concomitant medications when treatment with semaglutide started.

Type of Treatment	N = 117
**Antihyperglycemic Drugs**	
Insulin, *n* (%)	45 (38.5)
Total dose, units, median (IQR)	31.0 (20.0, 60.0)
Basal insulin, *n* (%)	45 (38.5)
Basal dose, units, median (IQR)	18.0 (14.0, 35.0)
Bolus insulin, *n* (%)	25 (21.4)
Bolus dose, units, median (IQR)	18.0 (12.0, 33.0)
**Oral antihyperglycemic drugs**	
Metformin, *n* (%)	83 (70.9)
Sulfonylurea, *n* (%)	14 (12.0)
iDPP-4, *n* (%)	29 (24.8)
Pioglitazones, *n* (%)	2 (1.7)
**Drugs with dual action on glycemia and weight**	
iSGLT2, *n* (%)	32 (27.3)
GLP-1 RA other than semaglutide *, *n* (%)	10 * (8.5)
**Antihypertensive drugs**	
ACE inhibitors, *n* (%)	75 (64.1)
Beta blockers, *n*/N (%)	18/116 (15.5) ^a^
Alpha blockers, *n* (%)	1 (0.8)
Diuretics, *n* (%)	42 (35.9)
Calcium channel blockers, *n* (%)	22 (18.8)
Potassium-conserving drugs, *n*/N (%)	2/115 (1.7) ^b^
**Lipid-lowering drugs**	
Statins, *n* (%)	50 (42.7)
PCSK9 inhibitors, *n* (%)	0 (0)
Fibrates, *n* (%)	8 (6.8)
Ezetimibe, *n* (%)	8 (6.8)
**Anticoagulant drugs, *n* (%)**	7 (6.0)
**Platelet-lowering drugs, *n* (%)**	21 (17.9)

* Liraglutide (7/10, 70.0%) and dulaglutide (3/10, 30.0%) were all suspended as soon as semaglutide therapy was initiated. ^a^ Results calculated over *n* = 116. ^b^ Results calculated over *n* = 115. ACE, angiotensin-converting enzyme; GLP-1 RA, glucagon-like peptide 1 receptor agonist; iDPP-4, dipeptidyl peptidase-4 inhibitor; IQR, interquartile range; iSGLT2, inhibitor of sodium-glucose cotransporter 2; PCSK9, proprotein convertase subtilisin/kexin type 9.

**Table 4:** Evolution of variables at 6 and 12 months after the start of semaglutide.

Variable	Basal Value (*n* = 117)	Δ 6 Months vs. Baseline	*n*	*p*	Δ 12 Months vs. Baseline	*n*	*p*
**Body weight** (kg)	102.5 (93.2, 116.1)	−9.3 (−13.8, −6.7)	117	<0.001	−11.20 (−17.00, −8.50)	83	<0.001
**FM** (kg)	47.75 (37.30, 55.15)	−7.55 (−11.18, −5.10)	116	<0.001	−9.70 (−13.10, −6.30)	61	<0.001
**FFM** (kg)	57.30 (49.35, 64.40)	−1.80 (−3.45, −0.10)	109	<0.001	−2.20 (−3.92, −0.77)	50	<0.001
**Visceral fat** (cm^2^)	240.10 (189.00, 261.10)	−32.90 (−49.30, −18.60)	119	<0.001	−37.70 (−62.13, −19.35)	58	<0.001
**SMM** (kg)	31.85 (26.60, 35.95)	−1.20 (−2.00, −0.20)	117	<0.001	−1.20 (−2.10, −0.60)	60	<0.001
**FMI** (kg/m^2^)	16.93 (13.47, 20.72)	−2.80 (−3.89, −1.94)	116	<0.001	−3.63 (−4.84, −2.42)	61	<0.001
**FFMI**	20.57 (18.73, 22.19)	−0.63 (−1.37, −0.03)	109	<0.001	−0.72 (−1.44, −0.29)	50	<0.001
**SMI** (kg/m^2^)	8.40 (7.70, 9.30)	−0.30 (−0.50, −0.10)	92	<0.001	−0.40 (−0.65, −0.10)	21	<0.001
**HbA1c** (%)	9.40 (7.90, 11.20)	−3.10 (−5.27, −1.80)	116	<0.001	−3.30 (−5.30, −1.50)	83	<0.001
**ECW** (L)	16.15 (14.35, 19.05)	−0.40 (−0.80, 0.0)	73	<0.001	−0.90 (−2.20, −0.02)	10	0.029
**ECW/TBW**	0.388 (0.379, 0.395)	0.001 (−0.003, 0.004)	91	0.043	0.0 (−0.003, 0.003)	30	0.409
**PhA** (^o^)	5.100 (4.500, 5.700)	−0.150 (−0.325, 0.125)	102	<0.001	−0.200 (−0.350, 0.100)	49	0.001

The results are expressed as the median (IQR). The one-tailed Wilcoxon test was used for comparison. HbA1c, glycosylated hemoglobin; ECW, extracellular water; FFM, fat-free mass; FFMI, fat-free mass index; FM, fat mass; FMI, fat mass index; IQR, interquartile range; PhA, phase angle SMM, skeletal muscle mass; SMI, skeletal muscle mass index; TBW, total body water.

**Table 5:** Changes in clinical and analytical parameters at 6 and 12 months versus baseline.

Variables	Δ 6 Months vs. Baseline	*n*	*p*	Δ 12 Months vs. Baseline	*n*	*p*
**Body weight** (kg)	−9.3 (−13.8, −6.7)	117	<0.001	−11.7 (−17.8, −8.7)	72	<0.001
**HbA1c** (%)	−3.10 (−5.27, −1.80)	116	<0.001	−3.70 (−5.27, −1.72)	72	<0.001
**BMI** (kg/m^2^)	−3.67 (−5.16, −2.39)	117	<0.001	−4.22 (−6.25, −2.89)	72	<0.001
**FBG** (mg/dL)	−90.0 (−171.0, −43.0)	95	<0.001	−96.0 (−143.3, −42.2)	54	<0.001
**eGFR** (mL/min/1.73 m^2^)	−1.0 (−7.7, 4.0)	84	0.049	0.0 (−11.0, 4.0)	47	0.156
**UACR** (mg/g)	−1.70 (−10.00, 2.40)	29	0.140	−5.10 (−21.23, 1.02)	10	0.042
**Albumin** (g/dL)	0.30 (−0.47, 0.77)	8	0.312	−0.10 (−0.32, 2.88)	4	0.427
**Creatinine** (mg/dL)	0.02 (−0.05, 0.11)	89	0.051	−0.01 (−0.07, 0.14)	51	0.195
**Uric acid** (mg/dL)	−0.20 (−0.90, 0.40)	27	0.052	−1.70 (−2.10, 0.0)	15	0.003
**Total cholesterol** (mg/dL)	−24.0 (−57.0, −3.5)	85	<0.001	−15.0 (−43.0, −1.0)	43	<0.001
**HDL** (mg/dL)	1.0 (−4.0, 4.0)	69	0.298	4.0 (0.0, 8.0)	31	<0.001
**LDL** (mg/dL)	−20.5 (−56.5, 2.7)	52	<0.001	−7.5 (−26.0, 6.2)	20	0.068
**Triglycerides** (mg/dL)	−64.5 (−94.2, −1.5)	86	<0.001	−43.0 (−133.0, −5.5)	45	<0.001
**SGPT** (U/L)	−11.0 (−33.5, −4.0)	81	<0.001	−11.0 (−29.0, −5.0)	37	<0.001
**SGOT** (U/L)	−9.0 (−25.5, −2.5)	33	<0.001	−10.5 (−25.2, −4.0)	16	<0.001
**FIB−4**	−0.21 (−0.42, 0.01)	29	0.003	−0.16 (−0.56, 0.05)	12	0.027

The results are Δ 6/12 months vs. baseline and are always expressed as the median (IQR). The one-tailed Wilcoxon test was used for comparison. BMI, body mass index; ECW, extracellular water; eGFR, estimated glomerular filtration rate; FBG, fasting blood glucose; FIB-4, fibrosis-4 score; HbA1c, glycosylated hemoglobin; HDL, high-density lipoprotein; IQR, interquartile range; ITT, intended-to-treat; LDL, low-density lipoprotein; SGOT, serum glutamic-oxaloacetic transaminase; SGPT, serum glutamate pyruvate transaminase; UACR, urine albumin-to-creatinine ratio.

## Data Availability

The data supporting the findings of this study are available upon reasonable request from the corresponding author.

## Supplementary Materials

The following supporting information can be downloaded at: https://www.mdpi.com/article/10.3390/jcm14155434/s1, Table S1: Body composition parameters assessed with MF-BIA.

## Abbreviations

T2D	Type 2 diabetes
HbA1c	Glycated hemoglobin
BW	Body weight
GLP-1 RAs	Glucagon-like peptide-1 receptor agonists
SGLT	Sodium-glucose linked transporter
SMF-BIA	Segmental multifrequency bioelectrical impedance analysis
BMI	Body mass index
FM	Fat mass
FMI	Fat mass index
VAT	Visceral adipose tissue
FFM	Fat-free mass
FFMI	Fat-free mass index
SMM	Skeletal muscle mass
EW	Extracellular water
TBW	Total body water
PhA	Phase angle
