# Supplementary material for: Once-Weekly Semaglutide Improves Body Composition in Spanish Obese Adults with Type 2 Diabetes: A 48-Week Prospective Real-Life Study
Source: J Clin Med. 2025 Aug 1;14(15):5434. doi: 10.3390/jcm14155434 (PMC12346962; doi:10.3390/jcm14155434)
Supplement: Supplementary file 1 [file jcm-14-05434-s001.zip › jcm-3575975-supplementary.pdf]

Supplementary Table S1. Body composition parameters assessed with MF-BIA

Parameter	Units	Definition
Total body Fat Mass (FM)	Kilograms	It is the portion of the human body that is composed strictly of fat.<br>It is composed by visceral fat and subcutaneous fat
Visceral Fat (VF)	Kilograms	Also known as intra-abdominal adipose tissue, it is the kind of fat stored within the abdominal cavity (it represents approximately 10% of the body fat mass).
Fat Mass Index (FMI)	Kilograms/square meters	It is the amount of fat mass relative to height. It is obtained by dividing fat mass by the square height.
Fat-Free Mass (FFM)	Kilograms	Body mass that comprises all body components except fat (body's water, organs, bone, and muscle content).
Fat-Free Mass Index (FFMI)	Kilograms/square meters	It is the amount of fat-free mass relative to height. It is obtained by dividing fat-free mass by the square height.
Skeletal muscle mass (SMM)	Kilograms	It refers to the total body's muscle tissue. It comprises approximately 40% of total body mass
Skeletal muscle mass index (SMMI)	Kilograms/square meters	It assesses muscle mass by dividing the area of all skeletal muscles on the cross-section of L3 vertebral body by the square of height.
Extracellular Water (EW)	Liters	It refers to the whole-body fluid outside the cells.
Total Body Water (TBW)	Liters	It is the total amount of fluid in the human body
EW/TBW Ratio		It represents the proportion of extracellular water related to total body water. It is associated with several diseases' prognosis.<br>A normal ECW/TBW ratio ranges between 0.360 and 0.390
Phase Angle (PhA)	Grades	It is the amount of reactance (cell membrane-specific resistance) divided by resistance (intracellular and extracellular resistance).<br>It is considered an indicator of cellular health, integrity and function.

MF-BIA, Multifrequency bioelectrical impedance analysis
